# The Interrater Reliability of the Greek Expanded and Revised Gross Motor Function Classification System and the Family Report Questionnaire in Cerebral Palsy

**DOI:** 10.3390/children13030368

**Published:** 2026-03-05

**Authors:** Vasileios C. Skoutelis, Renata Moutsiou, Maria Spanou, Zacharias Dimitriadis, Efstratia Kalamvoki, Vasiliki Zouvelou, Argyrios Dinopoulos

**Affiliations:** 1 Department of Physiotherapy, “ATTIKON” University General Hospital, 1 Rimini Str., 12462 Chaidari, Attica, Greece; vskoutelis@gmail.com (V.C.S.); renata.moutsiou@yahoo.com (R.M.); 2 Third Department of Pediatrics, “ATTIKON” University General Hospital, 1 Rimini Str., 12462 Chaidari, Attica, Greece; mariaier.spanou@gmail.com (M.S.); bzoubelou@gmail.com (V.Z.); argidino@yahoo.com (A.D.); 3 School of Medicine, National and Kapodistrian University of Athens, 75 Mikras Asias Str., 11527 Athens, Attica, Greece; 4 Department of Physiotherapy, School of Health Sciences, University of Thessaly, 3rd km of the Old National Road Lamia-Athens, 35132 Lamia, Central Greece, Greece; 5 “Paidokinisi” Pediatric Physiotherapy Practice, 18 Georgiou Papandreou Str., 16451 Argyroupolis, Attica, Greece; kalamvoki@gmail.com

**Keywords:** cerebral palsy, descriptors and illustrations, expanded and revised gross motor function classification system, family report questionnaire, interrater reliability

## Abstract

**Highlights:**

**What are the main findings?**

• The Greek-language GMFCS-E&R demonstrated substantial to excellent interrater reliability across pediatric neurologists, pediatric physiotherapists, and parents, with agreement strengthening in older age groups and reaching near-perfect levels between professionals.
• Most disagreements occurred between adjacent GMFCS levels, particularly IV/V and I/II, with parents more frequently assigning higher (more severe) levels than clinicians.

**What are the implications of the main findings?**

• The Greek GMFCS-E&R and GMFCS-FR are reliable tools for standardized classification in Greece, supporting consistent clinical decision-making, family-centered assessment, and alignment with international research standards.
• The findings demonstrate that the Greek GMFCS-E&R framework can be readily incorporated into everyday multidisciplinary care, enhancing communication among professionals and supporting coordinated service planning across clinical settings.

**Abstract:**

**Background/Objectives**: The Gross Motor Function Classification System–Expanded & Revised (GMFCS-E&R) is widely used to describe gross motor performance in children with cerebral palsy (CP). Although Greek-language materials are available, interrater reliability across healthcare professionals and parents has not been examined. This study evaluated the reliability of the Greek GMFCS-E&R among a pediatric neurologist, a pediatric physiotherapist, and parents, with an emphasis on descriptors and illustrations for older children and adolescents. **Methods**: A cross-sectional study was conducted with 111 children and adolescents with CP aged 2–18 years. Professionals classified each child using the Greek GMFCS-E&R brochure (ages 2–6) or the descriptors and illustrations (ages 6–12 and 12–18). Parents completed the age-appropriate GMFCS Family Report Questionnaire. Agreement among the three raters was assessed using Fleiss’ kappa (κ_F_), and pairwise agreement using weighted Cohen’s kappa (κ_Cw_), overall and by age band. **Results**: Overall interrater reliability was substantial (κ_F_ = 0.77). Agreement by GMFCS level ranged from κ_F_ = 0.68 (Level V) to κ_F_ = 0.85 (Level I). Reliability increased with age, reaching κ_F_ = 0.74–0.85 in adolescents. Pairwise agreement was excellent across all rater pairs, with near-perfect concordance between the pediatric neurologist and physiotherapist (κ_Cw_ = 0.98). In >60% of disagreements, parents assigned higher levels, typically between adjacent categories. **Conclusions**: The Greek-language GMFCS-E&R demonstrates high interrater reliability among healthcare professionals and parents, with excellent agreement when using descriptors and illustrations for older children and adolescents. The GMFCS-FR effectively incorporates parental perspectives and complements clinical assessment, supporting the use of the Greek GMFCS-E&R in routine clinical practice and research settings.

## 1. Introduction

Cerebral palsy (CP) is the leading cause of motor disability in childhood, with a birth prevalence of ~2 per 1000 live neonates in high-income countries, including Greece [1]. CP is not a single disease entity but a clinical umbrella term encompassing a group of permanent, but not unchanging, disorders of movement and posture caused by non-progressive disturbances in the developing brain, often accompanied by sensory, cognitive, communicative, behavioral, epileptic, and musculoskeletal problems [2].

To standardize the description and classification of motor impairment, the Gross Motor Function Classification System (GMFCS) was developed for children with CP up to 12 years, later expanded to 12–18 years and revised (GMFCS–Expanded & Revised, GMFCS-E&R) with updated 6–12 age-band descriptors and minor modifications throughout the original version. It classifies usual gross motor performance into five levels, ranging from Level I (independent walking without limitations) to Level V (transported in a manual wheelchair), across five age bands. The revision also incorporated environmental and personal factors (such as walking distances, energy demands, and social preferences) to improve developmental relevance [3,4]. Age-specific illustrations, aligned with GMFCS-E&R level descriptors, for the 6–12 and 12–18 bands enhance interpretation and conceptual clarity [5].

In line with the principles of inclusivity and shared decision-making, the GMFCS-E&R was adapted into the GMFCS Family Report Questionnaire (GMFCS-FR) [6,7]. This version enables parents, and where appropriate adolescents with CP, to actively participate in the classification. Available across four age bands, it promotes consistent application across languages and supports equitable care and international research collaboration [8].

Multiple language versions of the original GMFCS and GMFCS-E&R have shown excellent reliability. A recent meta-analysis [8] confirmed high agreement among healthcare professionals and between professionals and parents, with pooled intra-class correlation coefficients (ICCs) exceeding 0.90 for both versions. Validations span English [3,7], Korean [9], Turkish [10], Brazilian Portuguese [11,12], Chinese [13], Venezuelan Spanish [14], and Arabic [15]. These cross-cultural studies underscore the robustness of the GMFCS-E&R and the importance of confirming its stability across varied cultural and clinical contexts.

In Greece, the original GMFCS was previously validated by Papavasiliou et al. [16], demonstrating substantial agreement among pediatric neurologists. However, that study did not examine concordance with other healthcare professionals or parents. Although Greek-language GMFCS-E&R materials are available via the CanChild website (https://canchild.ca, accessed on 14 September 2025), no published data have assessed interrater agreement across multidisciplinary raters or in parent–professional comparisons. Addressing this gap is essential to confirm the reliability of the Greek version in clinical practice and to align national use with international standards. This need is particularly relevant given the updated descriptors for 6–12 years, the extension to 12–18 years, and the importance of professional–parent agreement in multidisciplinary, family-centered care.

This study aimed to evaluate interrater agreement between healthcare professionals and parents of children with CP (aged 2–18 years) using the Greek GMFCS-FR and to assess concordance between a pediatric neurologist and a pediatric physiotherapist applying standardized Greek-language GMFCS-E&R resources.

## 2. Materials & Methods

### 2.1. Participants

The study sample included children and adolescents aged 2 to 18 years diagnosed with CP, irrespective of subtype (spastic, dyskinetic, ataxic, or mixed) and topographical distribution (unilateral or bilateral, i.e., hemiplegic vs. diplegic/quadriplegic involvement). Participants were recruited through convenience sampling during scheduled visits to the Cerebral Palsy Outpatient Clinic (CPOC) of the Third Department of Pediatrics at ATTIKON University General Hospital, between January 2024 and July 2025. The CPOC provides multidisciplinary follow-up care to children from both the Athens metropolitan area and other regions of Greece, including Central Macedonia, Peloponnese, and Crete.

A priori sample size estimation for a 5 × 5 contingency table indicated that a minimum of 69 participants was required to achieve 80% statistical power at an alpha level of 0.05, assuming κ_1_ = 0.50 and κ_2_ = 0.70 [17]. A total of 111 children were ultimately recruited, exceeding the minimum requirement and thereby increasing confidence in the study findings.

Eligibility required sufficient linguistic and cognitive capacity in parents to comprehend written Greek. Written informed consent was obtained following oral and written explanation of the study. No exclusion criteria were applied beyond those described.

### 2.2. Measures

#### GMFCS-E&R

The GMFCS-E&R defines five levels of functional mobility across five developmental age bands: <2 years, 2–4 years, 4–6 years, 6–12 years, and 12–18 years. Classification is based on usual performance in everyday environments (home, school, community), emphasizing self-initiated movement, assistive technology, and contextual factors rather than capacity under standardized conditions [3,4].

Three official GMFCS-E&R resources supported classification. The GMFCS-E&R brochure provides textual descriptors for each level and is used by professionals based on clinical observation and/or review of medical records. The Greek-language version [18], formally approved by CanChild, was provided directly for this study but was not publicly available at the time of manuscript preparation.

As a complementary tool, the GMFCS-E&R descriptors and illustrations [5] offer visual representations for the 6–12 and 12–18 age bands. These illustrations serve as a practical alternative to the brochure and support faster, more intuitive classification by clarifying distinctions between adjacent levels and enhancing conceptual understanding [3,4].

The GMFCS-FR enables parents to classify their child’s motor abilities through structured self-report. Available for all age bands except for children < 2 years, it presents levels in descending order (V to I), prompting families to consider more severe limitations first. It uses accessible language and terminology tailored to non-clinicians [3,4].

The GMFCS-E&R demonstrates strong psychometric properties, with studies consistently reporting excellent inter- and intrarater reliability and high agreement between healthcare professionals and parents. Construct validity has been rated as strong, and content validity has been judged positive in the original development work [8].

All three tools were utilized in their official Greek-language versions. The GMFCS-E&R descriptors and illustrations, as well as the GMFCS-FR, were translated by the present authors in 2018 [19] and have been publicly available via the CanChild since that time.

### 2.3. Raters and Data Collection Procedure

For each participating child, three independent GMFCS-E&R ratings were completed by the child’s parent, a pediatric neurologist, and a pediatric physiotherapist.

Parents completed the age-appropriate GMFCS-FR based on personal knowledge, selecting the description that best represented their child’s abilities. Before participation, parents received the study information sheet, which included written instructions on how to complete the GMFCS-FR (e.g., “select the single description that best represents your child’s usual movement abilities”). Brief verbal instructions were also provided, and parents completed the questionnaire independently immediately after the clinical assessment. Adequate time was given.

The pediatric neurologist and physiotherapist independently assigned GMFCS levels at the end of the clinic visit, based on direct observation and/or discussion with the parent or child. For children aged 2–6 years, the Greek-language brochure was used; for ages 6–12 and 12–18, the descriptors and illustrations were applied.

Demographic data (child’s age, parental age, and education level) were self-reported; sex was retrieved from clinic records, and CP subtype was determined clinically by the pediatric neurologist. All data were recorded on coded paper forms to ensure anonymity and reviewed by the principal investigator for statistical analysis.

### 2.4. Data Analysis

Descriptive statistics were used to summarize the data. Measures of central tendency and dispersion were reported as means and standard deviations, respectively.

Interrater reliability was evaluated using complementary statistical methods, selected according to both the number of raters and the ordinal nature of the GMFCS-E&R classification. Fleiss’ kappa (κ_F_) was applied for agreement among all three raters [20,21]. For pairwise agreements between two raters, the linearly weighted Cohen’s kappa (κ_Cw_) coefficient was used [7,21]. To further refine the assessment of interrater reliability for ordinal data and to address potential limitations of kappa-based indices, Krippendorff’s Alpha (α_K_) was additionally calculated as a more robust agreement coefficient applicable to multiple raters and ordinal measurement levels [22].

Kappa values were interpreted using established thresholds: <0.20 poor, 0.21–0.40 fair, 0.41–0.60 moderate, 0.61–0.80 substantial, and 0.81–1.00 excellent agreement [21].

Krippendorff’s Alpha values were interpreted using established thresholds: ≥0.80 satisfactory, 0.67–0.79 tentative, and <0.67 poor agreement [22].

Interrater reliability analyses were conducted for the overall sample and separately by age band: 2–4 years, 4–6 years, 6–12 years, and 12–18 years.

Statistical significance was set at *p* = 0.05. Analyses were performed using IBM SPSS Statistics for macOS, version 29.0.2.0 (IBM Corp., Armonk, NY, USA). Krippendorff’s Alpha was computed using the “K-Alpha Calculator”, a freely accessible web-based application (https://www.k-alpha.org, accessed on 24 February 2026).

## 3. Results

### 3.1. Sample and Rater Profiles

A total of 111 children with CP participated (mean age = 8.9 years; SD = 4.6), 56% of whom were male. Most (91%) were diagnosed with spastic CP: 19 (17%) with unilateral (hemiplegia) and 82 (74%) with bilateral spastic CP (35 diplegia, 47 quadriplegia). Dyskinetic CP was identified in 9 children (8%), and one child (1%) had ataxic CP. Premature birth was reported in 67 children (60%), with a mean gestational age of 29.6 weeks (SD = 3.06; range = 24–36 weeks).

Parent respondents were predominantly mothers (70%), with a mean age of 42.16 years (SD = 8.03); 33% had tertiary education (Bachelor’s degree or higher). Although 111 children were enrolled, parental responses totaled 108, due to three sets of twins each represented by a single parent. Demographic and clinical characteristics are shown in Table 1.

Assessments were conducted by two licensed and specialized professionals: a pediatric neurologist (60 years old, 23 years of experience), and a pediatric physiotherapist (53 years old, 14 years of experience).

### 3.2. Interrater Reliability

Overall interrater agreement among the three raters was substantial (κ_F_ = 0.77; 95% confidence interval [CI]: 0.71–0.82). Agreement by GMFCS level ranged from substantial to excellent, with κ_F_ values from 0.68 (Level V) to 0.85 (Level I), all statistically significant (*p* < 0.001). Higher agreement was observed for Levels I-III (κ_F_ = 0.79–0.85), while Levels IV and V showed slightly lower values (κ_F_ ≈ 0.68) (Table 2).

Stratified analysis by age band further supported these findings. Agreement remained significant across all age groups, with κ_F_ values generally increasing with age and gross motor performance. In children aged 2–4 years, κ_F_ values ranged from moderate (0.54 for Level IV) to excellent (1.00 for Level I). For ages 4–6, κ_F_ values ranged from 0.47 (Level V) to 0.93 (Level II). In the 6–12 group, agreement remained substantial to excellent (κ_F_ = 0.53–0.93). The highest consistency was observed in adolescents (12–18 years), with κ_F_ values from 0.74 to 0.85 across all levels (*p* < 0.001) (Table 2).

Krippendorff’s Alpha analysis, applied to account for the ordinal nature of the GMFCS-E&R classification, demonstrated excellent overall interrater agreement among the three raters (α_K_ = 0.94; 95% CI: 0.91–0.96). This finding confirms the high level of interrater agreement observed across GMFCS-E&R classifications.

Pairwise agreement (κ_Cw_) indicated excellent reliability across all rater pairs. Agreement between the pediatric neurologist and physiotherapist was nearly perfect (κ_Cw_ = 0.98; *p* < 0.001). Agreement between the pediatric neurologist and parent (κ_Cw_ = 0.81) and between the pediatric physiotherapist and parent (κ_Cw_ = 0.82) was also excellent.

Only three disagreements occurred between the pediatric neurologist and physiotherapist: two between Levels IV and V and one between Levels II and IV. In contrast, 29 disagreements were recorded between the pediatric neurologist and parents: 13 between Levels IV and V, seven between Levels I and II, five between Levels II and III, two between Levels II and IV, one between Levels III and IV, and one between Levels III and V. In 22 of these cases (76%), parents assigned higher GMFCS levels, indicating less functional ability.

Similarly, 27 disagreements were recorded between the pediatric physiotherapist and parents: 12 between Levels IV and V, seven between Levels I and II, five between Levels II and III, and one each between Levels II and IV, III and IV, and III and V. In 17 of these cases (63%), parents also assigned higher levels (Table 3).

Most disagreements occurred between adjacent levels. Exceptions were rare and included six two-level discrepancies: four between Level II and Level IV (one neurologist vs. physiotherapist, one physiotherapist vs. parent, and two neurologist vs. parent) and two between Level III and Level V (both professionals assigned Level III, parent assigned Level V).

Age-stratified pairwise agreement revealed consistent patterns. Agreement between the pediatric neurologist and physiotherapist remained high across all age bands (κ_Cw_ ≥ 0.94; *p* < 0.001). Agreement between the pediatric neurologist and parent increased with age, from κ_Cw_ = 0.73 in the youngest group to 0.85 in adolescents. Similarly, agreement between the pediatric physiotherapist and parent ranged from substantial to excellent across all age bands (κ_Cw_ = 0.79–0.85; *p* < 0.001) (Table 3).

## 4. Discussion

This study provides the first validation of the Greek-language GMFCS-E&R and demonstrates that parents, pediatric neurologists, and pediatric physiotherapists can classify children’s gross motor function with high consistency. Agreement was particularly strong in children at Levels I–III, where mobility distinctions are clearer and everyday performance is easier to interpret. Lower agreement in Levels IV–V reflects the well-documented difficulty in differentiating between adjacent higher levels, where functional distinctions are more subtle and influenced by factors such as antigravity postural control, reliance on assistive technology, and the amount of physical assistance required. Many children at Levels IV and V also present overlapping functional profiles and often rely on similar mobility methods (being carried, pushed in a wheelchair, moving on the floor, or using powered mobility), with the relative use of each method varying across home, school, and community environments. Contextual influences, including access to assistive technology, environmental barriers, and family preferences, can further blur the distinction between the two levels. Consequently, small variations in usual performance or differences in how raters interpret functional support needs may influence whether a child is placed at Level IV or Level V. The tendency of parents to assign higher levels (indicating lower functional ability) than healthcare professionals likely indicates differences in perspective: families observe their children across multiple environments and activities, gaining a broader understanding of everyday performance that may not be fully captured during a single clinic visit. This broader familiarity can naturally lead to classifications that differ from those of healthcare professionals who rely primarily on structured observation [12,23,24].

These findings align with the earlier Greek validation of the original GMFCS [16], which reported κ_Cw_ = 0.80 between pediatric neurologists and noted that disagreements were predominantly observed at the II/III and IV/V boundaries. International evidence shows a similar pattern. Τhe meta-analysis by Piscitelli et al. [8] reported pooled ICCs of 0.94 for the original GMFCS and 0.96 for the GMFCS-E&R among healthcare professionals, and 0.93 and 0.90, respectively, for healthcare professional–parent agreement, supporting the strong reliability of the system across raters. Building on the previous Greek validation study [16], the present study expands the evidence base by incorporating both healthcare professionals and parents, extending the age range to 12–18 years, and applying updated descriptors and illustrations for older age bands.

To our knowledge, this is the first study in Greece to quantify healthcare professional–parent agreement using the GMFCS E&R in combination with the GMFCS-FR across a wide age range. The near-perfect agreement between the pediatric neurologist and physiotherapist highlights the influence of clinical expertise and familiarity with both the classification system and the children being assessed. Healthcare professional–parent agreement was likewise excellent and tended to increase with age, a pattern consistent with the study of McDowell et al. [7], who reported higher classification clarity and greater rater concordance in older children and adolescents.

Cross-cultural validations further contextualize these findings. The Danish version reported κ_Cw_ = 0.76–0.81 [25], the Thai version reported ICC = 0.90 [26], and the Gujarati version found κ_Cw_ = 0.84–0.91 for Levels I/V but substantially lower agreement for intermediate levels (κ_Cw_ = 0.54 for Level III; κ_Cw_ ≤ 0.25 for Levels II/IV) [27], illustrating parent–professional agreement ranging from fair to excellent, with parents often rating higher at adjacent boundaries, a pattern also evident in our sample. Similar findings have been reposted elsewhere. In Brazil, Silva et al. [12] found κ_Cw_ = 0.90 for therapist–therapist and κ_Cw_ = 0.71 for therapist–parent agreement. In China, Shi et al. [13] reported ICCs of 0.84–0.92 overall, with interprofessional ICCs of 0.86–0.92 in 6–12 year olds and 0.90–0.93 in adolescents, compared to parent–professional ICCs of 0.80–0.84. Jewell et al. [23] in Canada observed κ_Cw_ = 0.70 overall (κ_Cw_ = 0.73 in 4–6 year olds, κ_Cw_ = 0.66 in 2–4 year olds), with disagreements clustering at Levels III/IV and I/II. Together, these data reinforce two trends: agreement improves with age and adjacent GMFCS levels, notably I/II and IV/V; this poses classification challenges across languages and cultural contexts.

Evidence from other language versions also supports the robustness of the GMFCS E&R. The Turkish version [10] reported ICC = 0.97 between physiatrists; the Venezuelan version [14] found κ_Cw_ = 0.85–0.91 between examiners despite no prior exposure; and the Arabic version [15] showed κ_Cw_ = 0.80 between research and clinical physiotherapists, κ_Cw_ = 0.63 between parents and research physiotherapists, and κ_Cw_ = 0.57 between parents and clinical physiotherapists. In the Arabic study, parents tended to rate more highly when their child was under 4 years and lower in older children, a pattern attributed to developmental expectations in early childhood and functional gains or assistive device use in later years. This rater asymmetry is also reflected in our sample. Collectively, these findings confirm the cross-cultural reliability of the GMFCS-E&R and support the applicability of the Greek-language version under standardized administration.

The validation of the Greek GMFCS-E&R has clear implications for everyday clinical practice. A reliable Greek-language classification system enables healthcare professionals to describe gross motor function using a common, familiar language, supporting more consistent communication across disciplines. Its routine use can guide goal-setting, management planning, and timely referrals. The parallel validation of the GMFCS-FR further strengthens family-centered assessment by enabling parents to contribute observations from everyday environments, enhancing the decision-making process.

Limitations include the predominance of spastic CP, which may limit generalizability to rarer subtypes, and the single-site design. However, the CPOC at ATTIKON University General Hospital serves children from the Athens metropolitan area and other regions across Greece, including island communities, supporting its broader applicability. The sample was balanced across GMFCS levels, though the youngest age group (2–4 years) was relatively underrepresented (Ν = 12); still, the overall age distribution was adequate for stratified analyses. The <2 years band was not evaluated, as the GMFCS-FR applies from age 2; this band has already been validated in Greek for the original GMFCS [16], and descriptors remain unchanged in the E&R version. Finally, this study did not assess validity, test–retest reliability, or the GMFCS Self-Report Questionnaire for adolescents, which could provide additional perspectives.

## 5. Conclusions

The Greek-language GMFCS-E&R showed high interrater reliability between healthcare professionals and parents, with excellent agreement using descriptors and illustrations for older children and adolescents. The GMFCS-FR effectively integrates parental perspectives, complementing clinical assessment with insights from everyday performance. Consistent with international evidence, these results confirm the GMFCS-E&R’s cross-cultural applicability and support its use in clinical and research settings. Further studies should assess validity, test–retest reliability, and adolescent self-report integration to further strengthen the evidence base.

## Figures and Tables

**Table 1:** Descriptive information of the children and parents.

Children	Group (N = 111)
Age (years), mean (SD); range	8.9 (4.6); 2–18
Gender, N (%)	
male	62 (56)
female	49 (44)
CP type, N (%)	
Unilateral CP	19 (17)
Bilateral CP	82 (74)
Dyskinetic CP	9 (8)
Ataxic CP	1(1)
Parents	Group (N = 108)
Mother, N (%)	77 (71)
Father, N (%)	31 (29)
Age (years), mean (SD); range	42.16 (8.0); 23–62
Level of education, N (%)	
Primary education	8 (7)
Lower secondary education	8 (7)
Upper secondary education	37 (34)
Post-secondary non-tertiary education	11 (10)
Bachelor’s or equivalent level	33 (32)
Master’s or equivalent level	9 (8)
Doctoral or equivalent level	2 (2)

CP, cerebral palsy; N, number; SD, standard deviation.

**Table 2:** Interrater agreement among pediatric neurologist, physiotherapist, and parent across GMFCS-E&R levels I–V: overall sample and age bands.

			Fleiss’ Kappa	
		N *	Value	95% CI
Overall sample		111	0.77	0.71–0.82
GMFCS-E&R	I	20	0.85	0.74–0.96
	II	27	0.79	0.68–0.89
	III	20	0.84	0.74–0.95
	IV	26	0.69	0.58–0.80
	V	18	0.68	0.57–0.78
Age band: 2–4 years		12	0.72	0.55–0.89
GMFCS-E&R	I	3	1.00	0.67–1.33
	II	3	0.59	0.26–0.91
	III	3	0.65	0.32–0.97
	IV	1	0.54	0.21–0.86
	V	2	0.77	0.44–1.09
Age band: 4–6 years		27	0.77	0.65–0.89
GMFCS-E&R	I	2	0.84	0.63–1.06
	II	6	0.93	0.71–1.14
	III	5	0.82	0.60–1.04
	IV	12	0.74	0.53–0.96
	V	2	0.47	0.26–0.69
Age band: 6–12 years		35	0.73	0.63–0.83
GMFCS-E&R	I	5	0.75	0.56–0.94
	II	9	0.77	0.58–0.96
	III	6	0.93	0.74–1.12
	IV	7	0.53	0.34–0.72
	V	8	0.69	0.50–0.89
Age band: 12–18 years		37	0.79	0.70–0.89
GMFCS-E&R	I	10	0.85	0.67–1.04
	II	9	0.78	0.59–0.97
	III	6	0.85	0.67–1.04
	IV	6	0.74	0.55–0.92
	V	6	0.74	0.55–0.92

* Participant counts (N) per GMFCS-E&R level reflect the most frequently assigned classification across the three independent raters for each individual, with the prevailing rating adopted as the final level. CI, confidence interval; GMFCS-E&R, Gross Motor Function Classification System–Expanded & Revised.

**Table 3:** Distribution of GMFCS-E&R ratings and pairwise interrater agreement among raters: overall sample and age bands.

		GMFCS-E&R Levels						Cohen’s Weighted Kappa	
Overall Sample (N = 111)		I	II	III	IV	V	Total	Value	(95% CI)
	Pediatric Physiotherapist								
Pediatric Neurologist	I	20	0	0	0	0	20		
	II	0	27	0	1	0	28		
	III	0	0	20	0	0	20		
	IV	0	0	0	25	1	26		
	V	0	0	0	1	16	17		
	Total	20	27	20	27	17	111	0.98	0.95–1.00
	Parent								
Pediatric Neurologist	I	15	5	0	0	0	20		
	II	2	24	0	2	0	28		
	III	0	5	13	1	1	20		
	IV	0	0	0	15	11	26		
	V	0	0	0	3	14	17		
	Total	17	34	13	21	26	111	0.81	0.75–0.87
	Parent								
Pediatric Physiotherapist	I	15	5	0	0	0	20		
	II	2	24	0	1	0	27		
	III	0	5	13	1	1	20		
	IV	0	0	0	16	11	27		
	V	0	0	0	3	14	17		
	Total	17	34	13	21	26	111	0.82	0.76–0.88
Age band: 2–4 years (N = 12)									
	Pediatric Physiotherapist								
Pediatric Neurologist	I	3	0	0	0	0	3		
	II	0	3	0	0	0	3		
	III	0	0	3	0	0	3		
	IV	0	0	0	1	1	2		
	V	0	0	0	0	1	1		
	Total	3	3	3	1	2	12	0.94	0.84–1.04
	Parent								
Pediatric Neurologist	I	3	0	0	0	0	3		
	II	0	2	0	1	0	3		
	III	0	2	1	0	0	3		
	IV	0	0	0	1	1	2		
	V	0	0	0	0	1	1		
	Total	3	4	1	2	2	12	0.73	0.46–0.99
	Parent								
Pediatric Physiotherpist	I	3	0	0	0	0	3		
	II	0	2	0	1	0	3		
	III	0	2	1	0	0	3		
	IV	0	0	0	1	0	1		
	V	0	0	0	0	2	2		
	Total	3	4	1	2	2	12	0.79	0.54–1.04
Age band: 4–6 years (N = 27)									
	Pediatric Physiotherapist								
Pediatric Neurologist	I	2	0	0	0	0	2		
	II	0	6	0	0	0	6		
	III	0	0	5	0	0	5		
	IV	0	0	0	12	0	12		
	V	0	0	0	0	2	2		
	Total	2	6	5	12	2	27	1.00	1.00–1.00
	Parent								
Pediatric Neurologist	I	2	0	0	0	0	2		
	II	1	5	0	0	0	6		
	III	0	0	3	1	1	5		
	IV	0	0	0	8	4	12		
	V	0	0	0	0	2	2		
	Total	3	5	3	9	7	27	0.79	0.64–0.93
	Parent								
Pediatric Physiotherapist	I	2	0	0	0	0	2		
	II	1	5	0	0	0	6		
	III	0	0	3	1	1	5		
	IV	0	0	0	8	4	12		
	V	0	0	0	0	2	2		
	Total	3	5	3	9	7	27	0.79	0.64–0.93
Age band: 6–12 years (N = 35)									
	Pediatric Physiotherapist								
Pediatric Neurologist	I	5	0	0	0	0	5		
	II	0	9	0	1	0	10		
	III	0	0	6	0	0	6		
	IV	0	0	0	6	0	6		
	V	0	0	0	1	7	8		
	Total	5	9	6	8	7	35	0.95	0.87–1.02
	Parent								
Pediatric Neurologist	I	3	2	0	0	0	5		
	II	1	8	0	1	0	10		
	III	0	1	5	0	0	6		
	IV	0	0	0	2	4	6		
	V	0	0	0	1	7	8		
	Total	4	11	5	4	11	35	0.80	0.69–0.91
	Parent								
Pediatric Physiotherapist	I	3	2	0	0	0	5		
	II	1	8	0	0	0	9		
	III	0	1	5	0	0	6		
	IV	0	0	0	3	5	8		
	V	0	0	0	1	6	7		
	Total	4	11	5	4	11	35	0.82	0.73–0.91
Age band: 12–18 years (N = 37)									
	Pediatric Physiotherapist								
Pediatric Neurologist	I	10	0	0	0	0	10		
	II	0	9	0	0	0	9		
	III	0	0	6	0	0	6		
	IV	0	0	0	6	0	6		
	V	0	0	0	0	6	6		
	Total	10	9	6	6	37	37	1.00	1.00–1.00
	Parent								
Pediatric Neurologist	I	7	3	0	0	0	10		
	II	0	9	0	0	0	9		
	III	0	2	4	0	0	6		
	IV	0	0	0	4	2	6		
	V	0	0	0	2	4	6		
	Total	7	14	4	6	6	37	0.85	0.76–0.93
	Parent								
Pediatric Physiotherapist	I	7	3	0	0	0	10		
	II	0	9	0	0	0	9		
	III	0	2	4	0	0	6		
	IV	0	0	0	4	2	6		
	V	0	0	0	2	4	6		
	Total	7	14	4	6	6	37	0.85	0.76–0.93

CI, confidence interval; GMFCS-E&R, Gross Motor Function Classification System–Expanded & Revised; N, number.

## Data Availability

The datasets presented in this article are not readily available because of ethical and technical limitations. Requests to access the datasets should be directed to the corresponding author.
